# DNA recovery and STR profiling from heated tobacco sticks for forensic personal identification

**DOI:** 10.1007/s12024-025-01151-z

**Published:** 2026-01-15

**Authors:** Sara Amurri, Giulia Fazio, Filippo Pirani, Arianna Giorgetti, Susi Pelotti, Carla Bini

**Affiliations:** Department of Medical and Surgical Sciences, Unit of Legal Medicine, University of Bologna, Via Irnerio 49, Bologna, 40126 Italy

**Keywords:** Forensic genetics, Cigarette butts and filters, DNA recovery, STR profiling, Personal identification

## Abstract

“Heated tobacco products” (HTPs), featuring a heating blade placed either in the holder (HH) or within the stick itself (EH), are increasingly being used. The composition and mode of consumption, along with the possible reduction in salivation induced by their use, could affect DNA recovery and STR profiling. The aim of this work was to assess the DNA recovery useful for personal identification from two commercially available types of HTP sticks, compared with traditional cigarettes. Sixteen volunteers each smoked 2 traditional cigarettes, 2 HTP sticks of type HH and 2 HTP sticks of type EH each (*n* = 96). For each type, one filter paper was analyzed immediately (T0), and another after one month of storage in a controlled environment (T1). DNA amounts ranged from 0.265 to 7.4294 ng/μl for traditional cigarettes and 0.0008 ng/μl to 6.4711 ng/μl for HTP sticks, showing a significant difference (*p* = 0.0092). DNA recovery from traditional cigarette, but not from HTP sticks, was significantly lower at T1 compared to T0. Ninety-two samples (95.8%) were amplified, yielding 88 full STR profiles (95.6%), 4 partial profiles (4.4%) and 5 mixed profiles (5.4%), while no profiles deemed inconclusive. Mixed profiles likely resulted from persistent DNA transferred prior to the controlled conditions. Heated tobacco sticks may lead to lower DNA recovery than traditional cigarettes, potentially impacting other forensic DNA analyses that require higher DNA yield than STR profiling.

**Supplementary Information:**

The online version contains supplementary material available at 10.1007/s12024-025-01151-z.

## Introduction

At crime scenes, various types of biological traces useful for reconstructing events and for personal identification of involved individuals can be found. Saliva, for example, may be found on various surfaces, such as cigarette butts, which can serve as important DNA sources in forensic cases. Indeed, these samples can contain DNA from saliva, mouth epithelial cells, and the smoker's latent fingerprints [1, 2].

In recent years, new and alternative tobacco products, such as "heat-not-burn" (HNB) or heated tobacco products (HTPs) have been introduced [3]. These products typically consist of a battery-powered tobacco heating holder used in combination with a disposable tobacco stick. The heating process occurs at lower temperatures than conventional combustible cigarettes, usually around 350 °C, although some devices may reach up to 550 °C [4]. Consequently, HTPs are purported to be less harmful than traditional cigarettes. Due to strict anti-smoking regulations in many countries worldwide, the use of HTPs, either as an alternative or complement to traditional products, has experienced global growth in recent years, with significant adoption in the Asia–Pacific region and growing interest in Europe and North America [5]. Current HTPs use is higher among males (3.45%) than females (1.82%) [6], with projections suggesting further increases in the next decade [4]. This rising trend is likely to be reflected in crime scenes investigations and have implications for forensic science, as it increases the likelihood of HTPs being collected as evidence at crime scenes. The global adoption of tobacco alternatives also raises questions regarding their sampling methods and DNA recovery from their filter papers. Despite their widespread use, to the best of the author’s knowledge, no studies have specifically addressed these aspects.

The structural characteristics of HTPs may also impact forensic DNA analysis, given the availability of different HTP models, with the most common configurations involving a heating element housed either in the holder or inside the tobacco stick [4]. Moreover, varying temperatures reached during use could potentially affect DNA integrity.

The increasing use of HTP devices has led researchers to investigate their effects on human health, including oral health, inflammatory responses and immune mediators. A recent study showed that HTP users may have lower saliva and salivary enzymes secretion rates compared to traditional cigarette smokers and non-smokers [7]. However, other studies have reported similar salivary flow rates between traditional cigarettes users and HTP users [8, 9].

A possible reduction in salivary secretion could negatively affect the deposition of DNA on HTP sticks and DNA recovery for STR profiling, which may require evaluating the impact on forensic investigations, such as potential changes in sampling methods.

The purpose of the present research was to assess DNA recovery, useful for personal identification, from the filter papers of two types of commercially available HTP sticks, compared with traditional cigarette, as well as to evaluate the impact of the time interval between smoking and DNA analysis.

## Materials and methods

### Experimental design and sample collection

The study was conducted in compliance with ethical standards, was approved by the Bioethical Committee of the University of Bologna (Prot. n. 0175936 approved on 29.06.2023). Volunteers who agreed to participate in the research project were asked to fill out and sign the informed consent form. Sixteen volunteers, 8 men and 8 women, aged 22 to 63 years (mean age 31.9 years, standard deviation 13.9, median age 24.5 years, interquartile range 23–39.5), were recruited among habitual smokers and were left free to smoke with no predefined or standardized timing (including interval between device use and duration of smoking) or puffing regimen.

Each volunteer provided 6 samples by smoking 2 traditional cigarettes, 2 tobacco sticks with a heating holder (HTP stick type HH) and 2 tobacco sticks featuring an embedded heating blade (HTP stick type EH) during the same day, leading to a total of 32 samples per treatment and 96 samples collected overall. To avoid inter-day variability smoking sessions took place on the same day, with at least with an interval of thirty minutes between one another.

Immediately following their use, each cigarette butt and stick was placed by the volunteer in a disposable plastic bag, using one bag per type of cigarette. The procedure was conducted such that only the smoker handled the cigarette butt or stick.

Each sample was handled with sterile forceps to remove and cut half of the filter paper using a sterile razor blade before DNA extraction (see Supplementary material Figure A).

Out of the 2 traditional cigarettes and tobacco sticks smoked by each person per type, one filter paper was extracted immediately after the consumption (T0), and one was stored for 1 month at room temperature in a controlled environment, to avoid contamination before the extraction process (T1). The sampling procedure and the filter paper size was the same between traditional cigarettes and heated tobacco products.

From volunteers, the buccal swabs were collected by a sterile dry cotton swab (Copan Italia S.p.A., Italy) as reference samples. After collection, the swabs were stored at − 20 °C until further processing.

### Genotyping

#### DNA extraction and quantification

Samples were extracted using the Maxwell^®^ FSC DNA IQ™ Casework kit (Promega corporation) on Maxwell^®^ 16 RSC instrument (Promega corporation, Madison, WI, USA), following the Extraction of Samples on a Solid Support with CW Spin Basket protocol according to manufacturer’s recommendations. Each DNA extract was eluted in 50 µl of Elution Buffer. Quality and quantity of 2 µl of extracted DNA, including the presence of inhibitors, degradation index, and DNA mixtures, were determined using the PowerQuant^®^ System (Promega corporation, Madison, WI, USA) in a final reaction volume of 20 µl.

#### DNA amplification and STR analysis

Ninety-two out of 96 samples with DNA quantification values ≥ 6 pg/µl (threshold determined by internal laboratory validation) were amplified using 5 µl reaction volume of GlobalFiler™ IQC PCR Amplification Kit (Thermo Fisher Scientific, Waltham, MA, USA) with 29 cycles on the Veriti™ 96-Well Thermal Cycler (Applied Biosystems, Thermo Fisher Scientific, Waltham, MA, USA) instrument. PCR products were separated and detected by capillary electrophoresis on SeqStudio™ Genetic Analyzer (Applied Biosystems, Thermo Fisher Scientific, Waltham, MA, USA) following the manufacturer instructions. GlobalFiler™ Allelic Ladder was included in each capillary electrophoresis run. Data collection and GeneMapper™ ID-X v 1.6 softwares (Thermo Fisher Scientific, Waltham, MA, USA) were respectively used to collect raw data and for allele calls using an analytical threshold value of 100 RFU.

### Data interpretation and statistical analysis

The interpretation of electrophoretic data was carried out according to the national Ge.F.I. recommendations [10]. The generated DNA profiles were classified into full profiles, with 21 correctly typed loci, or partial profiles, showing a lower number of correctly typed loci. Moreover, DNA profiles were classified as single source profiles, with ≥ 10 STR loci successfully amplified and characterized by no more than two alleles at each locus, and mixed profiles, with ≥ 10 STR loci successfully amplified with more than two alleles detected in at least two different loci. To complete the profile outcomes, mixed profiles were then classified as mixed profiles with a major contributor, when one or two alleles at each locus were in a ratio of peak height ≥ 3:1 relative to the other alleles of the same locus. On the contrary, when the allele peak height ratio was < 3:1, profiles were classified as mixed with no major contributor [11, 12].

DNA profiles were compared to the reference samples of the donors, to evaluate the match and the occurrence of locus and allelic dropout, consisting of missing alleles at one or more genetic loci. To assess the value of the DNA match, the likelihood ratio (LR) was calculated using the biostatistical evaluation by LRmix Studio software v. 2.1.5, after estimating the dropout probability. LR values above the level of 10^6^ were considered for donor identification [10].

Descriptive statistics was provided for all data. To check for a normal distribution, the Stata sk-test, that inherently takes into consideration skewness and kurtosis, was used, followed by non-parametric statistic tests.

DNA amounts yielded by men and women for each cigarette type and sampling time (T0 and T1) were compared using a non-parametric t-test (Mann–Whitney test). Volunteers were also categorized into two age groups (≤ 30 or > 30 years), and DNA yields for each cigarette type and sampling time were compared between these groups by Mann–Whitney test.

The comparison of DNA amounts and degradation index among the three types of cigarettes (traditional, HTP stick type HH and HTP stick type EH) was performed using the Friedman test, a non-parametric ANOVA for paired samples, followed by a post hoc Dunn’s multiple comparison test. The analysis for paired samples allows each volunteer to be compared with themselves, reducing the influence of individual smoking behavior on the results.

To assess the impact of the timing factor on DNA recovery and degradation index, DNA levels at T0 and T1, for each cigarette type, were compared using the Wilcoxon matched-pairs signed-rank test, a non-parametric test for paired samples.

Additionally, the Chi-square test was applied to assess the associations between cigarette type and profile outcome (full profile vs partial, and single source vs mixed profiles) at both T0 and T1.

In all statistical analyses, obtained using Stata/MP 15.1, the significance level was set at < 0.05, while GraphPad Prism version 8.2.1 was used to create graphs.

## Results

Overall, DNA values yielded from traditional cigarettes samples ranged from a minimum of 0.2650 ng/μl to a maximum of 7.4294 ng/μl, while those recovered from the tobacco sticks ranged from 0.0008 ng/μl to 6.4711 ng/μl (see Table 1 for median and interquartile (IQ) ranges). Since the differences between men and women were not statistically significant (*p* > 0.05), the two subpopulations were combined into a single group. Similarly, comparisons between the age groups (≤ 30 or > 30 years) for each cigarette type and sampling time tested not statistically significant (*p* > 0.05).

**Table 1:** Results of DNA quantification for traditional cigarettes and heated tobacco products (HTP) sticks, type HH (tobacco sticks with a heating holder) and type EH (tobacco sticks featuring an embedded heating blade)

	DNA quantification median (IQ range) [ng/μl]	
Timing	*T0*	*T1*
Traditional cigarettes	0.5427 (0.3662–1.9415)	0.3834 (0.1709–0.6686)
Heated tobacco products stick with a heating holder (type HH)	0.1540 (0.0152–0.7271)*	0.1547 (0.0145–1.1123) †
Heated tobacco products stick type with an embedded heating blade (type EH)	0.3520 (0.0667–0.7130)*	0.1237 (0.0831–0.4782)

**Fig. 1:**
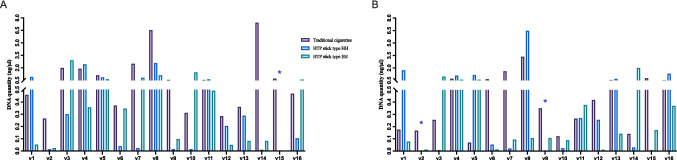
DNA quantification results by single volunteers 1–16 (v1-v16) with traditional cigarette, HTP stick type HH and HTP stick type EH, at T0 (Figure A) and at T1 (Figure B). The* y* axis was split in order to provide a magnified view of the samples with DNA between 0 and 0.5 pg/µl. * = samples (HTP stick type EH and type EH, from v2, v9 and v15) that yielded DNA amounts < 6 pg/µl

DNA quantification results obtained from samples analyzed at T0 are provided in Fig. 1. These results show high interindividual as well as intraindividual variability.

Background control samples did not show quantifiable DNA (data not shown). No inhibitors were present in all tested samples for the subsequent PCR amplification step, according to the PowerQuant^®^ kit and to the GlobalFiler™ IQC PCR Amplification Kit results. Additionally, the PowerQuant^®^ System provided a degradation index ranging from 3.4 to 4.5 in 3 out of 96 samples (3.13%), two of which were HTP stick type EH at T1, and one was HTP stick type HH at T1.

**Fig. 2:**
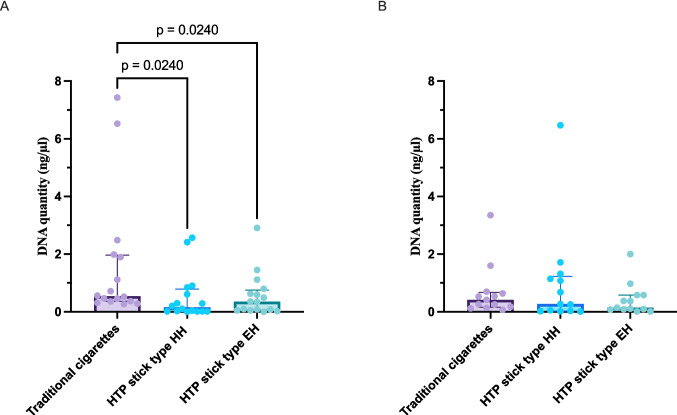
In A and B, comparison of DNA quantities recovered from traditional cigarette, HTP stick type HH and HTP stick type EH, considering only samples extracted at T0 in A and those extracted at T1 in B. p values are shown for statistically significant comparisons

The comparison among traditional cigarettes, HTP stick type HH and HTP stick type EH using the Friedman test showed a statistically significant difference in DNA yield at T0 (*p* = 0.009). Post-hoc Dunn’s multiple comparison confirmed that traditional cigarettes yielded a significantly higher amount of DNA compared to both types of HTP (both *p* = 0.024), as shown in Fig. 2A. This result was confirmed significant even after excluding potential outliers from the analysis (*p* = 0.017). In contrast, when considering only samples extracted after one month of storage (T1), the difference in DNA quantity between traditional cigarettes and HTP sticks was not statistically significant (*p* = 0.269) (Fig. 2B).

No statistically significant difference was found in the degradation index among traditional cigarettes, HTP stick type HH, and HTP stick type EH filters when compared at the same time points, both at T0 and at T1 (*p* > 0.05) (comparison of different types at fixed times, T0 or T1).

**Fig. 3:**
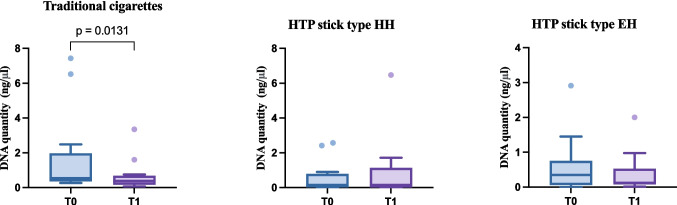
DNA quantities yielded from traditional cigarette (**A**), HTP stick type HH (**B**) and HTP stick type EH (**C**), shown as comparison of T0 vs T1. HTP: heated tobacco products. HH: tobacco sticks with a heating holder; EH: tobacco sticks featuring an embedded heating blade. p values are shown for statistically significant comparisons

The amount of DNA recovered from traditional cigarettes at T1, after 1 month of storage in a protected environment, was significantly lower compared to samples immediately processed at T0 (*p* = 0.013). On the contrary, the Wilcoxon test showed no statistically significant difference between T0 and at T1 for HTP stick type HH and HTP stick type EH filters (*p* = 0.252 and *p* = 0.211, respectively). Results are shown in Fig. 3.

**Fig. 4:**
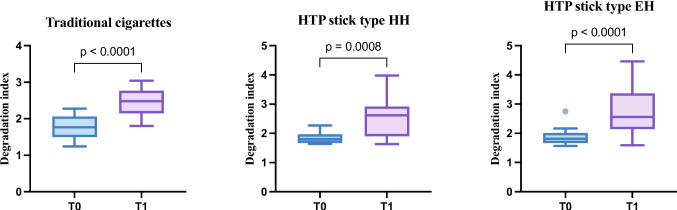
Degradation index of DNA from traditional cigarette, HTP stick type HH and HTP stick type EH, shown as comparison of T0 vs T1. HTP: heated tobacco product. HH: tobacco sticks with a heating holder; EH: tobacco sticks featuring an embedded heating blade. p values are shown for statistically significant comparisons

When comparing degradation index values over time within each cigarette type, it appeared significantly higher (*p* = 0.001) in all samples extracted at T1 compared to those extracted at T0, with *p* = 0.001 for traditional cigarettes, *p* = 0.001 for HTP stick type HH and *p* = 0.001 for HTP stick type EH tobacco filters, as shown in Fig. 4 (comparison of different timing at fixed each type).

From the 96 total samples, 92 samples (95.8%) were above the threshold value of 0.006 ng/μl and were then selected and carried out for the amplification.

From the selected 92 samples, 32 consisted of traditional cigarette filters (16 processed at T0 and 16 at T1), 29 of HTP stick type HH filters (15 at T0 and 14 at T1), and the other 31 cigarette butts were HTP stick type EH filters (15 processed at T0 and 16 at T1). Results are shown in Table 2.

**Table 2:** Genotyping results showing, for traditional cigarettes, HTP stick type HH and HTP stick type EH, and for each timing, T0 and T1, the number of amplified samples and the outcome of STR profiling

	Timing	Amplified samples	Full profiles	Partial profiles	Single source profiles	Mixed profiles		Profiles allowing donor identification
						With no major contributor	With major contributor	
Traditional cigarettes	T0	16	16		15	1		16
	T1	16	16		15		1	16
HTP stick type HH	T0	15	15		13	1	1	15
	T1	14	11	3	14			14
HTP stick type EH	T0	15	15		15			15
	T1	16	15	1	15		1	16
*Total*		***92 (95.8%)***	***88 (95.6%)***	***4 (4.4%)***	***87 (94.6%)***	***2***	***3***	***92 (100%)***

Full STR profiles were obtained in 88 samples (95.6%), partial profiles in 4 (4.4%), and no profile was deemed inconclusive.

Eighty-seven samples were classified as single source profiles, and 5 as mixed profiles, of which 2 (2.3% of full profiles) without a major contributor and 3 (13.2% of full profiles) with a major contributor. Mixed profiles, one originating from a female subject and four from male subjects, showed a maximum number of alleles/locus ≤ 4. Partial and mixed profiles were characterized by stochastic effects, such as allelic and locus drop-out, and/or allelic imbalance, that were particularly noticeable in larger-sized loci, such as SE33.

In Table 2, the number of samples amplified for traditional cigarettes and HTP sticks and the resultant DNA profiles are reported.

Considering the profiles outcomes obtained from T0 and T1 samples, a slight but not statistically significant reduction of full profiles at time T1 was observed for both HTP sticks tobacco filters, type HH and type EH, where locus dropout was observed in 9% of samples. Overall, the profiles outcomes did not show a statistically significant association with the tested variables (full vs partial profiles or single vs mixed profiles at T0 or T1).

The results of the statistical analysis performed using the LRmix Studio v. 2.1.5 software showed for full or partial single-source profiles a LR range of 10^20^ < LR < 10^30^ and for mixed profiles a LR range of 10^14^ < LR < 10^27^. The percentage of profiles allowing the identification of the donor with LR ≥ 10^6^ was 100%.

## Discussion

Considering the widespread increase in the use of alternative HTP devices for smoking [3, 5, 6] and the likelihood of encountering them at crime scenes, the present study aimed to assess the impact of new HTPs sticks on DNA recovery and STR profiling for personal identification purposes.

The quantification results, as shown by the large interquartile range and individual values depicted in Fig. 1, demonstrated high inter and intra-individual variability in DNA yield. However, it should be noted that previous studies have shown large variations in the quantity of DNA obtained from saliva samples from the same donor at different times of day [13], which may be reflected in our data.

Given the wide range of DNA amounts observed in our results and the variability between individuals, we questioned the reliability of values at the extremes of the range. We first focused on four samples with DNA yields of less than 6 pg/µl, all provided by three volunteers who smoked HTP sticks (v2, v9, v15 in Fig. 1 A and B). Notably, one volunteer contributed two samples, one of type EH and one of type HH, both analyzed at T0 (v15 in Fig. 1A). The other two volunteers each provided one sample with DNA amounts of less than 6 pg/µl; both were recovered from HTP type EH and analyzed at T1 (Table 1, and v2 and v9 in Fig. 1B). However, these 3 volunteers were not considered poor shedders since their corresponding traditional cigarette samples yielded higher DNA concentration. Therefore, their data were retained in the statistical analyses.

Conversely, at the upper end of the range, two samples from traditional cigarettes provided by two volunteers (v8 and v14 in Fig. 1A) showed DNA amounts higher than 6.5 ng/μl at T0, confirmed by DNA re-analysis. The DNA quantification results from the corresponding HTP sticks were then examined, and only one of the two volunteers (v8) also exhibited high DNA levels in all samples. The other (v14) only showed such high DNA amount in one sample, so the corresponding sample was considered an outlier.

Our results indicate higher DNA recovery from traditional cigarettes compared to both types of HTP stick, HH and EH, when considering samples extracted immediately after smoking (T0). This difference may be attributed to variations in user behaviour patterns (also called puffing topography) between traditional cigarettes and HTP sticks, as has been shown for e-cigarettes [14]. For example, prolonged contact with the mouth or extended handling of traditional cigarettes could result in greater deposition of biological material onto the filter paper. However, this hypothesis cannot be verified because smoking behaviour was not standardized in the present study, which represents a limitation of the study we will further discuss in the limitations section.

Another possible explanation lies in the reduction of salivary flow induced by the new HTP sticks [7], which could decrease the amount of biological material deposited by the smoker on the sticks.

Conversely, the different configurations of the heating element between HH and EH sticks, and the corresponding temperatures they reach (not measured), do not appear to impact DNA recovery, with no PCR inhibition or significant degradation observed at T0. Nevertheless, it should be noted that traditional cigarettes usually reach even higher temperatures, which does not prevent forensic DNA profiling, as demonstrated by our quantification results.

To overcome the lower DNA recovery from HTP sticks, analyzing DNA recovered from both halves of the filter paper could be considered in the forensic setting, although typically only one half of the filter paper is analyzed to prioritize sample preservation for further analyses.

The quantification results showed that traditional cigarette filter papers extracted at T1, one month after smoking, yielded lower DNA levels compared to samples extracted at T0, along with a significantly higher degradation index. The timing factor did not impact DNA recovery for HTP sticks, as the difference in DNA quantities between T0 and T1 was not statistically significant, and degradation indices for HTP sticks were not higher at T1 than at T0. Our results can be explained by the fact that DNA degradation can occur through enzymatic, non-enzymatic degradation, oxidative damage, and interaction with chemical agents also present in natural compounds and industrial chemicals, and these effects can persist over time [15]. However, it should be noted that in the present study, storage was conducted in a controlled environment, while more realistic conditions or outdoor storage could have a greater impact.

Four stick samples extracted at T1 generated partial STR profiles; however, these profiles were still suitable for comparison with the reference, with a number of typed loci ≥ 10, allowing donor identification (LR > 10^6^).

Except for these four samples, high-quality and stochastic effects-free genetic profiles were obtained, despite the statistically lower amounts of DNA recovered from HTP sticks compared to traditional cigarettes. Indeed, all calculated LR values were higher than 10^6^, and were therefore deemed useful for personal identification.

On the other hand, DNA genotyping in our study revealed mixed profiles in five samples, likely due to indirect DNA transfer events [16] or contamination with environmental DNA (eDNA) [17]. Possible scenarios for the presence of exogenous DNA on cigarette butts and HTP sticks include personal activities such as intimate contact before handling and smoking, or aerial transfer of DNA from clothing, skin, and other items onto butts and filters without previous contact, making interpretation of results more complicated.

The present work has some limitations, the foremost being the design with volunteers smoking three different devices on the same day – a behaviour that might seem unlikely. However, recent surveys in the United States showed that approximately half of surveyed adults use more than one electronic smoking device, and the co-use of traditional cigarette and e-cigarettes is common [18, 19]. Moreover, given the large number of variables affecting shedder status, including day-to-day fluctuations, we aimed at minimizing inter-day variability in individual shedding propensity [20].

As already mentioned, another limitation might be the lack of a fully controlled design regarding variables such as the order of smoking, intervals between device use and duration of smoking for each device, given that these factors can influence the DNA deposition. On the other hand, since smokers typically exhibits individual gestures, timing, and behaviour [21], we used statistical tests for paired data, that allow intra-subject comparisons across different cigarettes and devices. We believe this approach could control for personal variability, theoretically reducing the influence of individual smoking style and focusing the comparison solely on the effect of the cigarette or device type.

That said, the lower quantity of DNA yielded by HTP sticks compared to traditional cigarettes, especially in samples with DNA amounts below the optimal DNA input suggested in the literature, could affect the application and outcome of other DNA analyses, such as forensic DNA phenotyping (FDP) for age estimation [22, 23] and rapid DNA analyses [24], which are useful investigative tools but typically require higher DNA amounts.

## Conclusions

Modern HTPs, which may increasingly be collected at crime scenes over time, can result in lower DNA recovery than traditional cigarettes – regardless of the heating element configuration – whether embedded in the holder or in the stick. The reduced DNA recovery might be due to decreased salivary flow or different smoking behaviour, rather than temperature, which is usually lower in HTP sticks compared to the combustion process. Nevertheless, all amplified HTP samples allowed identification of the donor, suggesting that HTP sticks should be collected and sampled in the same way as traditional cigarettes, as they are useful items for DNA recovery and STR profiling.

## Key points

1. DNA recovery was assessed from heated tobacco products (HTP) compared to cigarettes.
2. DNA recovery from HTP sticks was significantly lower than from traditional cigarettes.
3. Time affected DNA recovery from cigarettes, but not from HTP sticks.
4. HTP sticks allowed DNA profiling, but may limit other forensic DNA analyses.
5. Forensic genetics must consider emerging items that may be found at crime scenes.

## Supplementary Information

Below is the link to the electronic supplementary material.

Supplementary file1 (PDF 2238 KB)
