# Supplementary material for: DNA recovery and STR profiling from heated tobacco sticks for forensic personal identification
Source: Forensic Sci Med Pathol. 2026 Jan 15;22(2):512–20. doi: 10.1007/s12024-025-01151-z (PMC13331821; doi:10.1007/s12024-025-01151-z)

Supplementary material to: **DNA recovery and STR profiling from heated tobacco sticks for forensic personal identification**

Figure A. Example of the sampling method from traditional cigarettes

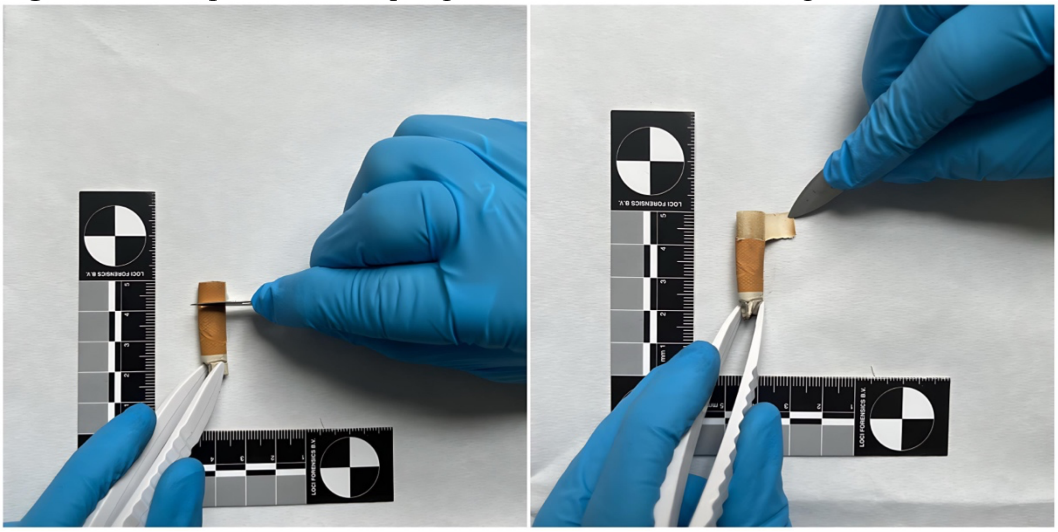

Figure B. Example of the sampling method from heated tobacco products (HTPs) type HH.

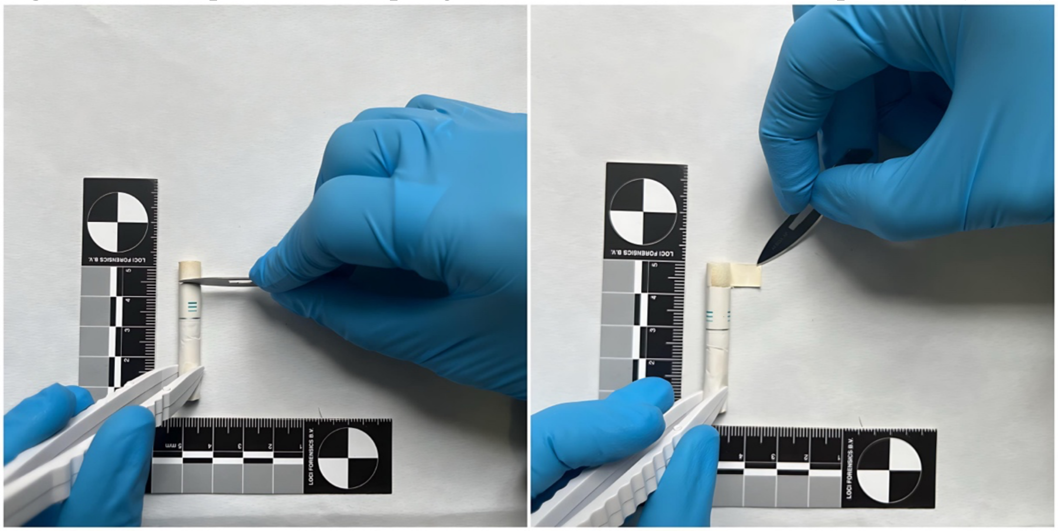

Figure C. Example of the sampling method from heated tobacco products (HTPs) type EH.

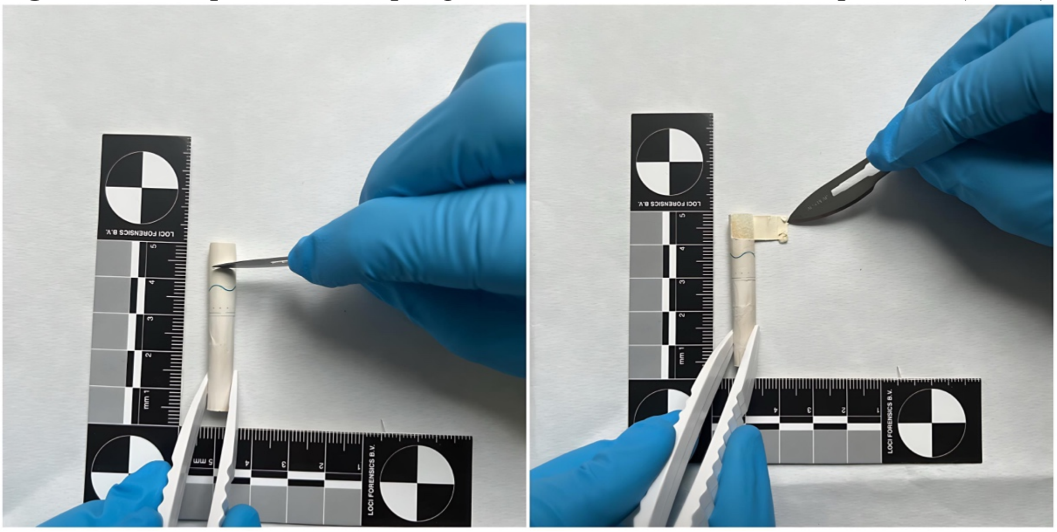

Supplement: Supplementary file 1 — Supplementary file1 (PDF 2238 KB) [file 12024_2025_1151_MOESM1_ESM.pdf]
